# Prenatal Aflatoxin B_1_ Exposure: A Review of Pathogenesis and Impact on Fetal Skeletal Development and Ossification

**DOI:** 10.3390/toxins18030122

**Published:** 2026-03-01

**Authors:** Giovana Perez Montenegro, João Victor Batista da Silva, Sher Ali, Sana Ullah, Lucas Gabriel Dionisio Freire, Carlos Augusto Fernandes de Oliveira, Leandra Náira Zambelli Ramalho

**Affiliations:** 1 Department of Pathology and Forensic Medicine, School of Medicine of Ribeirão Preto, University of São Paulo, Ribeirao Preto 14049-900, SP, Brazil; giovana.montenegro@usp.br (G.P.M.); joaovsilva@usp.br (J.V.B.d.S.); 2 Department of Food Engineering, School of Animal Science and Food Engineering, University of São Paulo, Pirassununga 13635-900, SP, Brazil; alisher@usp.br (S.A.); sanaullah@usp.br (S.U.); lucasgdfreire@usp.br (L.G.D.F.)

**Keywords:** AFB_1_, prenatal exposure, skeletal development, ossification defects, oxidative stress

## Abstract

Prenatal exposure to aflatoxin B_1_ (AFB_1_) poses a significant risk to fetal development and is associated with reduced birth weight in humans. Experimental studies consistently show that AFB_1_ induces fetal abnormalities, with skeletal malformations and ossification defects being the most common. However, the specific impact of AFB_1_ on fetal osteogenesis remains unclear. Given this knowledge gap, this study aimed to review the existing literature concerning the pathogenesis of AFB_1_ and its potential influence on bone development. The primary mechanisms implicated in AFB_1_’s impact on bone include dysfunction in vitamin D and calcium metabolism, alterations in parathyroid hormone production and function, induction of inflammatory responses, and oxidative stress. Collectively, these mechanisms have the potential to impair osteoblast and osteoclast function and, consequently, compromise ossification. Based on these findings, studies should explore and elucidate the effects of AFB_1_. Elucidating these mechanisms is crucial for mitigating the deleterious impacts of AFB_1_ on fetal skeletal development.

## 1. Introduction

Globally, an estimated 14.7% of infants are born with low birth weight each year [1]. Additionally, approximately 15 million preterm births and 2 million stillbirths occur annually, while 12–15% of clinically confirmed pregnancies up to 20 weeks’ gestation result in spontaneous abortion [2,3]. Together, these adverse gestational outcomes are major causes of maternal and fetal morbidity and mortality.

Among the environmental contributors to these outcomes, mycotoxin exposure during pregnancy represents a significant yet frequently underrecognized risk factor. One of the most potent and prevalent mycotoxins is aflatoxin B_1_ (AFB_1_), produced by *Aspergillus* species that contaminate staple foods such as maize and groundnuts [4,5]. Warm climates, high humidity, and inadequate storage conditions promote *Aspergillus* proliferation and AFB_1_ biosynthesis, resulting in disproportionately high exposure in tropical and subtropical regions [5,6].

Importantly, AFB_1_ crosses the placental barrier and directly affects the fetus [7]. Animal studies consistently demonstrate that gestational AFB_1_ exposure results in a broad spectrum of fetal toxicities, including growth restriction, immunotoxicity, and organ-specific teratogenic effects [8,9,10]. In humans, epidemiological studies indicate that maternal biomarkers of AFB_1_ exposure correlate with reduced birth weight and impaired fetal growth [11,12,13].

Among the fetal outcomes associated with prenatal AFB_1_ exposure, skeletal abnormalities including delayed ossification, reduced mineralization, and structural malformations have been frequently reported across several experimental models [14,15]. Yet, despite the consistency of these findings, the pathways by which AFB_1_ perturbs fetal bone development remain incompletely defined. Multiple biological processes appear to be involved, but their interactions and relative contributions during gestation are not well delineated.

This review aims to synthesize current evidence on AFB_1_ pathogenesis with an emphasis on its potential role in disrupting fetal skeletal development. To provide conceptual clarity, this study examines the mechanisms of AFB_1_ toxicity and describes the relationship of prenatal exposure to AFB_1_ with skeletal development, calcium homeostasis, hormonal modulation, oxidative stress-related cytotoxicity, and inflammatory pathways. Through this integrated framework, the study identifies the central mechanistic themes that may underlie AFB_1_-induced fetal bone deficits and outlines key hypotheses for future investigation.

## 2. Aflatoxins

Aflatoxins are a group of mycotoxins produced as secondary metabolites, including AFB_1_, AFB_2_, AFG_1_, and AFG_2_, synthesized predominantly by fungi of the genus *Aspergillus*, particularly *Aspergillus flavus* and *A. parasiticus* [16,17]. These mycotoxins are frequent contaminants of a wide range of food commodities and animal feeds. Chemically, aflatoxins are difuranocoumarin derivatives, characterized by a highly conjugated coumarin nucleus fused to a bifuran system, which accounts for their physicochemical and structural distinctions. Among them, AFB_1_ exhibits the greatest toxic, mutagenic, and carcinogenic potential [4,18]. Structurally, AFB_1_ varies from AFB_2_ by the presence of an unsaturated double bond in the terminal furan ring, a feature that enhances its chemical reactivity and underlies its increased biological toxicity. AFB_1_ contamination of food crops results from a combination of pre-harvest and post-harvest factors, including climatic conditions that favor fungal proliferation, such as high temperature, drought stress, and humidity, as well as inadequate harvesting, transportation, and storage practices [17,19]. Insect damage and elevated post-harvest moisture further facilitate fungal colonization and toxin accumulation. These environmental pressures activate the aflatoxin biosynthetic gene cluster in *Aspergillus* spp., promoting secondary metabolite production and increasing AFB_1_ levels in susceptible crops [17,19]. Climate change has intensified these risk factors, contributing to the expanding geographic distribution and prevalence of aflatoxin contamination.

AFB_1_ occurs in a broad range of staple foods, including cereals and oilseeds, making dietary intake the primary route of human exposure, including during pregnancy through maternal consumption [13,16,20]. Following ingestion, AFB_1_ undergoes hepatic bioactivation, where cytochrome P450 enzymes convert it into a highly reactive 8,9-epoxide intermediate capable of forming covalent adducts with DNA and proteins. As a result, the global burden of AFB_1_ exposure is substantial, with several billion individuals estimated to be chronically exposed to levels exceeding recommended safety thresholds [21].

High-dose exposure to aflatoxins can result in acute aflatoxicosis, characterized by severe and potentially fatal hepatotoxicity [22]. This acute toxicity is attributed to the electrophilic nature of reactive metabolites and instability of the lactone ring, leading to rapid hepatocellular necrosis, impaired coagulation, and liver failure. Notably, outbreaks such as the 2004 aflatoxicosis episode in Kenya, linked to consumption of heavily contaminated maize, illustrate the severity of acute exposure and its associated clinical manifestations, including hepatic necrosis and coagulopathy [23]. In contrast, chronic low-dose exposure, particularly to AFB_1_, is strongly associated with hepatocellular carcinoma, owing to its genotoxic and mutagenic properties. Based on extensive epidemiological, experimental, and mechanistic evidence, AFB_1_ is classified as a Group 1 carcinogen by the International Agency for Research on Cancer (IARC) [24].

### 2.1. AFB_1_ General Mechanism of Action

AFB_1_ toxicity is critically dependent on hepatic bioactivation mediated by cytochrome P450 (CYP450) enzymes, predominantly CYP1A2 and CYP3A4 [25]. Through this metabolic conversion, AFB_1_ is transformed into several metabolites, among which AFB_1_-exo-8,9-epoxide represents the most biologically reactive and toxic species. This electrophilic epoxide readily forms covalent adducts with cellular macromolecules, particularly DNA and proteins, thereby initiating mutagenic and carcinogenic processes [26]. The interaction of AFB_1_-exo-8,9-epoxide with DNA leads predominantly to the formation of AFB_1_–N^7^–guanine adducts, which, if not efficiently repaired, result in GC→TA transversions. A hallmark outcome of this mutagenesis is the TP53 codon 249 (R249S) mutation, frequently detected in hepatocellular carcinoma cases from regions with high aflatoxin exposure [27].

Beyond genotoxicity, AFB_1_ induces pronounced oxidative stress, a key contributor to its cytotoxic and carcinogenic effects. This occurs via enhanced generation of reactive oxygen species (ROS) during CYP-mediated metabolism [28], coupled with the depletion of intracellular antioxidant defenses, including glutathione (GSH) and the enzymatic activities of superoxide dismutase (SOD) and catalase [29,30]. Sustained oxidative stress promotes lipid peroxidation, mitochondrial dysfunction, and activation of redox-sensitive signaling pathways, amplifying hepatocyte injury and favoring malignant transformation. These processes also sensitize cells to inflammatory signaling and apoptotic dysregulation. AFB_1_ additionally interferes with protein synthesis, particularly during acute exposure. The toxin forms covalent complexes with RNA molecules, impairing transcriptional fidelity and ribosomal function, ultimately leading to inhibition of translation, cellular degeneration, and necrosis [30,31].

Moreover, AFB_1_ activates pro-inflammatory signaling cascades, characterized by increased expression of cytokines such as tumor necrosis factor-α (TNF-α) and interleukin-1α (IL-1α) [24,32]. This inflammatory milieu can contribute to chronic liver injury, fibrosis, and tumor promotion. Collectively, these interrelated mechanisms, including genotoxicity, oxidative stress, impaired protein synthesis, and inflammation, form the mechanistic basis by which AFB_1_ exerts systemic toxicity, including during prenatal development. Detailed discussion of oxidative stress– and inflammation-mediated skeletal effects is provided in subsequent Section 2.6 and Section 2.7 to avoid redundancy.

### 2.2. AFB_1_ and Prenatal Exposure

Experimental studies consistently demonstrate that prenatal exposure to AFB_1_ produces a range of developmental toxicities in animal models. Among the most frequently reported outcomes are skeletal abnormalities, including delayed ossification, reduced bone mineralization, and structural malformations [10,15]. These skeletal effects often occur alongside visceral toxicity, reduced fetal and neonatal body weight, and a decrease in litter size, reflecting both direct embryotoxicity and compromised gestational viability [9]. Additional findings include alterations in reproductive organ development, which may impair fertility and reproductive efficiency in adulthood [33], as well as neurobehavioral changes, suggesting disruption of central nervous system development following in utero exposure [8].

**Figure 1:**
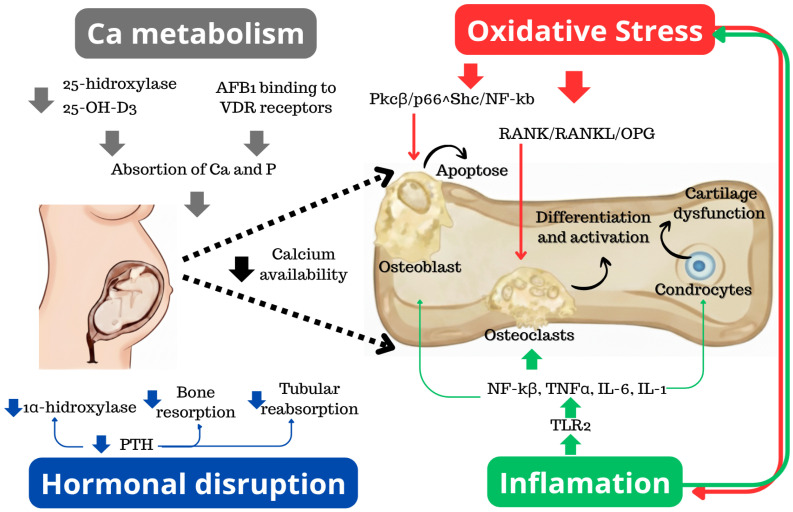
Integrated mechanisms of AFB_1_-induced fetal bone toxicity. The framework highlights four core causal nodes: (1) Oxidative Stress, (2) Inflammation, (3) Hormonal Disruption and (4) Calcium Metabolism. Maternal AFB_1_ exposure disrupts calcium metabolism and hormonal regulation through impaired vitamin D activation, altered parathyroid hormone signaling, and reduced calcium and phosphorus availability. Concurrent activation of oxidative stress and inflammatory pathways in fetal skeletal tissues promotes osteoblast apoptosis, enhances osteoclast differentiation via RANK/RANKL/OPG signaling, and induces chondrocyte dysfunction, collectively leading to delayed ossification and impaired skeletal mineralization. PTH: parathyroid hormone; Ca: calcium; P: phosphorus; NF-κB: nuclear factor kappa B; TNF-α: tumor necrosis factor-α; IL-6: interleukin-6; IL-1: interleukin-1; TLR2: Toll-like receptor 2; 25-hydroxylase: an enzyme converting vitamin D to 25-hydroxyvitamin D; 25-OH-D_3_: 25-hydroxyvitamin D_3_; PKCβ/p66^Shc/NF-κB: protein kinase C β/p66^Shc/nuclear factor kappa B signaling pathway; RANK/RANKL/OPG: receptor activator of nuclear factor κB/receptor activator of nuclear factor κB ligand/osteoprotegerin; VDR: vitamin D receptor.

The integrated mechanistic relationships linking maternal exposure, placental dysfunction, and fetal skeletal injury are summarized in Figure 1. Epidemiological studies in human populations further support these experimental observations. Maternal exposure to AFB_1_ during pregnancy has been associated with lower birth weight, reduced length, and impaired postnatal growth trajectories, as assessed through biomarker-based exposure analyses [11,12,34]. These outcomes are particularly evident in regions with high dietary aflatoxin burden. Evidence of aflatoxin–albumin adducts in cord blood confirms that AFB_1_ and its metabolites can cross the placental barrier and directly reach the fetus during critical windows of organogenesis [7]. Recent advances in biomarker-based exposure assessment and placental transfer characterization further support the likelihood of direct fetal tissue exposure, reinforcing the relevance of emerging studies examining skeletal-cell–specific responses to AFB_1_. In addition to direct fetal exposure, prenatal toxicity may arise indirectly through maternal hepatic dysfunction, oxidative stress, systemic inflammation, and impaired nutrient and endocrine regulation, which together can disrupt placental function, calcium homeostasis, and intrauterine development. Accordingly, prenatal AFB_1_ toxicity should be interpreted within a unified maternal–placental–fetal framework. Following maternal dietary exposure, hepatic bioactivation of AFB_1_ induces systemic oxidative, inflammatory, metabolic, and endocrine disturbances that compromise placental transport capacity and alter the intrauterine redox and cytokine environment. Impaired fetal osteogenesis due to intrauterine exposure to AFB1 can occur due to four main mechanisms outlined in Figure 1. The first refers to oxidative stress (1), leading to osteoclastic activation and osteoblast apoptosis. As a result of this process, there is bone matrix degradation and decreased osteogenesis. Next, strictly related to oxidative stress, inflammation (2) is stimulated, and in addition to the damage related to oxidative stress, there is also dysfunction of the growth cartilage, compromising fetal bone growth. From a metabolic point of view, another observed mechanism is the impairment of parathyroid hormone (3), which directly interferes, leading to a reduction in the availability of calcium to be used in osteogenesis, but also further influences the fourth affected mechanism, which is calcium metabolism (4). In this case, there is also an additional loss of circulating calcium accessibility, definitively impairing fetal osteogenesis [35,36,37]. Importantly, current human evidence remains largely limited to associations with fetal growth restriction, whereas mechanistic understanding derives predominantly from animal and cellular studies, underscoring the need for cautious translational interpretation.

### 2.3. AFB_1_ and Skeletal Development

The skeletal system originates during embryogenesis primarily from the mesoderm, specifically the paraxial mesoderm and lateral plate mesoderm, with additional contributions from the neural crest [38]. Skeletal elements derived from the paraxial mesoderm and lateral plate mesoderm give rise predominantly to the axial and appendicular skeleton, including the long bones, whereas neural crest–derived mesenchyme contributes mainly to the craniofacial skeleton and flat bones of the skull [38,39]. Only developmental features directly relevant to ossification vulnerability are emphasized here to maintain focus on AFB_1_-related skeletal toxicity.

Bone formation is a highly regulated, multistep process involving coordinated cellular proliferation, differentiation, migration, and extracellular matrix deposition, orchestrated by tightly regulated signaling pathways and transcription factors [40]. In humans, skeletal development initiates during early fetal life and continues through postnatal growth, with complete skeletal maturation typically achieved between 25 and 27 years of age [41].

Two principal ossification mechanisms govern skeletal development. Intramembranous ossification, responsible for the formation of most flat bones, including the cranial vault and clavicles, involves the direct differentiation of mesenchymal stem cells into osteoblasts within highly vascularized connective tissue [42]. In contrast, endochondral ossification, which underlies the formation of long bones and vertebrae, proceeds through the establishment of a cartilaginous template. This template undergoes chondrocyte proliferation, hypertrophy, apoptosis, and subsequent replacement by bone-forming osteoblasts derived from invading osteoprogenitor cells [43]. Primary ossification centers develop within the diaphysis during fetal life, followed by the formation of secondary ossification centers in the epiphyses after birth. Although the general sequence of ossification is conserved, the timing and progression of these events vary substantially among species, an important consideration when interpreting animal-model data [34,44].

Among the most consistently reported outcomes of prenatal AFB_1_ exposure, ossification defects are prominent and widely documented across animal species. These alterations include delayed or incomplete ossification of cranial and long bones, absence or malformation of bony processes, and hypoplasia of the axial skeleton (Table 1). Such defects suggest that AFB_1_ interferes with critical stages of both intramembranous and endochondral ossification.

Although the precise mechanisms by which AFB_1_ disrupts fetal bone development remain incompletely defined, several plausible pathways can be inferred from its known toxicological properties. AFB_1_-induced oxidative stress, DNA damage, and inflammatory signaling may impair the proliferation and differentiation of osteogenic and chondrogenic progenitor cells, while hepatic dysfunction and placental insufficiency may indirectly compromise fetal mineral availability and endocrine regulation. Experimental evidence indicates that oxidative stress can suppress osteoblast activity and delay matrix mineralization [49], leading to phenotypes consistent with those observed following AFB_1_ exposure. Notably, skeletal development is highly sensitive to disturbances in calcium homeostasis and hormonal signaling, including vitamin D, parathyroid hormone, and sex steroids, all of which may be altered secondary to AFB_1_ exposure.

### 2.4. AFB_1_ and Calcium Metabolism

Vitamin D plays a key role in calcium and phosphorus homeostasis, both of which are indispensable for normal bone mineralization. Vitamin D_3_ (cholecalciferol), obtained through dietary intake or synthesized in the skin under ultraviolet B radiation, undergoes hepatic hydroxylation to form 25-hydroxycholecalciferol (25-OH-D_3_). This metabolite is subsequently converted in the kidney by 1α-hydroxylase (CYP27B1) into the biologically active hormone 1,25-dihydroxycholecalciferol (1,25-(OH)_2_D_3_), which exerts its effects through binding to the vitamin D receptor (VDR) [50]. Activated VDR functions as a ligand-dependent transcription factor that regulates genes involved in intestinal calcium and phosphate absorption, including *TRPV6*, *SLC34A2*, and *CALB1*, thereby maintaining skeletal mineral balance [50,51,52].

AFB_1_ disrupts calcium metabolism through multiple, converging mechanisms that impair mineral availability for bone formation. First, AFB_1_ interferes with hepatic vitamin D metabolism by altering the activity of vitamin D 25-hydroxylase, thereby reducing the conversion of cholecalciferol to 25-OH-D_3_ [53]. Second, experimental evidence indicates that AFB_1_ can interact with VDR signaling, competitively inhibiting ligand binding and/or modulating receptor-mediated transcriptional activity, leading to dysregulated expression of calcium and phosphorus transport genes [54]. Notably, exposure to AFB_1_ has been associated with a state of functional vitamin D deficiency, wherein circulating 25-OH-D_3_ concentrations may remain within normal ranges while downstream vitamin D–dependent signaling is impaired [55]. This phenomenon highlights the importance of receptor functionality and intracellular signaling, rather than serum vitamin D levels alone, in maintaining calcium homeostasis. In line with these molecular disruptions, animal studies have demonstrated that AFB_1_ exposure results in hypocalcemia, impaired bone mineralization, and reduced bone mineral density, reinforcing the link between AFB_1_ toxicity and compromised skeletal integrity [56].

During fetal development, the fetus relies entirely on maternal calcium and phosphorus supply, which is actively transported across the placenta to support rapid skeletal growth [57]. Prenatal exposure to AFB_1_ may therefore compromise fetal mineralization both directly, by impairing placental calcium transport mechanisms, and indirectly, through maternal vitamin D dysfunction and hypocalcemia. These disorders can adversely affect both endochondral and intramembranous ossification, resulting in delayed ossification center formation, structural abnormalities, and persistence of non-mineralized bone regions [15,24,58]. Because calcium homeostasis is tightly integrated with endocrine regulation, particularly involving parathyroid hormone (PTH) and vitamin D signaling, hormonal disturbance represents an additional mechanism through which AFB_1_ intensifies skeletal developmental defects, as discussed below.

### 2.5. AFB_1_ and Hormonal Disruption

Bone metabolism is regulated by a complex endocrine network in which PTH plays a pivotal role. Secreted by the parathyroid glands in response to declining serum calcium levels, PTH stimulates osteoclastic bone resorption, enhances renal calcium reabsorption, and promotes activation of vitamin D through upregulation of renal *CYP27B1*, thereby increasing intestinal calcium absorption [59]. Experimental and toxicological studies indicate that AFB_1_ exposure disrupts calcium homeostasis and the functional PTH–vitamin D axis, leading to reduced calcium mobilization and persistent hypocalcemia [55]. Importantly, available evidence suggests that these effects arise indirectly, as a consequence of hepatic and renal toxicity, oxidative stress, and impaired vitamin D metabolism, rather than through direct suppression of PTH synthesis or receptor antagonism. Oxidative stress and organ dysfunction induced by AFB_1_ may secondarily impair endocrine regulation of calcium balance, potentially affecting calcium sensing and hormone responsiveness. However, direct effects of AFB_1_ on parathyroid tissue, calcium-sensing receptor (CaSR) signaling, or PTH receptor (PTH1R) activity have not been conclusively demonstrated and remain hypothetical [60,61]. During pregnancy, maternal calcium homeostasis is tightly regulated to meet fetal demands, with increased intestinal absorption and mobilization from maternal bone serving as key compensatory mechanisms. AFB_1_-induced hypocalcemia and endocrine dysregulation may therefore reduce placental calcium transfer, directly compromising fetal skeletal mineralization and contributing to delayed ossification and bone hypomineralization [57].

### 2.6. AFB_1_ and Oxidative Stress: Destruction of Osteoblasts and Osteoclasts

Oxidative stress is defined as a pathological imbalance between the production of reactive oxygen species (ROS) and the capacity of cellular antioxidant defense systems to neutralize them [62]. During pregnancy, excessive oxidative stress has been linked to fetal growth restriction, developmental abnormalities, and fetal demise, reflecting the heightened vulnerability of the developing organism [63].

Following hepatic bioactivation, AFB_1_ forms reactive epoxide intermediates that covalently bind to DNA and proteins. At the mitochondrial level, AFB_1_ disrupts membrane potential and electron transport, resulting in increased ROS generation and activation of the intrinsic apoptotic pathway via caspase-9 and caspase-3 [64]. In addition, AFB_1_ interferes with complex I of the mitochondrial respiratory chain and undergoes redox cycling during metabolism by cytochrome P450 enzymes, leading to excessive formation of hydrogen peroxide (H_2_O_2_) and hydroxyl radicals (•OH) [65]. Concomitantly, AFB_1_ compromises endogenous antioxidant defenses by depleting reduced GSH and suppressing antioxidant enzyme activity, while also perturbing the arachidonic acid cascade, thereby amplifying oxidative stress and promoting inflammatory signaling [66,67].

In immune and metabolically active cells, AFB_1_ alters the expression of genes involved in oxidative phosphorylation, increasing ROS and malondialdehyde (MDA) levels while reducing intracellular GSH content [68]. Additional studies have shown that AFB_1_ decreases superoxide dismutase (SOD) activity, further impairing redox homeostasis [69]. Oxidative stress is a well-established modulator of bone metabolism, capable of damaging osteoblasts and osteoclasts, disrupting intracellular signaling, inducing apoptosis, and ultimately impairing bone remodeling [70].

In osteoblasts, elevated ROS activate the PKCβ/p66^Shc^/NF-κB signaling pathway, triggering apoptosis and stimulating the release of pro-inflammatory cytokines, including tumor necrosis factor-α (TNF-α), interleukin-1β (IL-1β), and interleukin-6 (IL-6) [71]. This process reduces osteoblast viability and shifts bone remodeling toward resorption. Oxidative stress–induced skeletal alterations also involve Forkhead box O (FoxO) transcription factors, which regulate genes associated with antioxidant defense and cellular survival [72]. Genetic studies provide strong evidence for the role of FoxO proteins in bone homeostasis. In mice, deletion of FoxO1, FoxO3, and FoxO4 resulted in decreased osteoblast number and reduced bone formation in both trabecular and cortical compartments, whereas overexpression of FoxO3 attenuated oxidative stress, enhanced osteoblast survival, and increased bone mass [73]. Similarly, FoxO1 knockout mice exhibited reduced expression of osteogenic markers, an effect reversed by treatment with N-acetylcysteine, underscoring the central role of oxidative stress in osteoblast dysfunction [74]. Excess ROS also promotes osteoclastogenesis, stimulating differentiation and activation of osteoclasts through the RANK/RANKL/OPG axis, an effect amplified by ROS-induced activation of mitogen-activated protein kinases (MAPKs) and NF-κB signaling [75].

Although direct evidence linking AFB_1_-induced oxidative stress to fetal bone pathology remains limited, extrapolation from developmental biology suggests heightened vulnerability during gestation. Fetal skeletal tissues undergo intense proliferation, differentiation, and remodeling, creating a critical window of susceptibility to redox imbalance. In laboratory mammals, antioxidant enzyme activity increases markedly during late gestation [76]. In humans, SOD activity in erythrocytes rises by approximately 17 weeks of gestation, whereas glutathione peroxidase activity remains comparatively low even after birth, indicating reduced antioxidant capacity during fetal life [77]. This developmental imbalance may render fetal bone cells particularly sensitive to oxidative injury. Physiological levels of ROS also serve as signaling molecules during embryogenesis, regulating transcription factors involved in proliferation, differentiation, and apoptosis [78]. However, excessive ROS disrupt these tightly controlled processes. In mouse models, oxidative stress altered placental expression of osteogenic genes, including *Hgf*, *Kitl*, and *Il1b*, resulting in skeletal malformations [79]. Together, these findings suggest that AFB_1_-induced oxidative stress can impair fetal bone development both directly, by damaging osteogenic cells, and indirectly, by activating inflammatory pathways, as described.

### 2.7. AFB_1_ and Inflammation

Inflammation exerts a profound influence on bone metabolism, primarily by modulating the RANK/RANKL/OPG signaling pathway, which governs osteoclast differentiation and activity [75,80,81]. Pro-inflammatory cytokines such as TNF-α and IL-1β activate NF-κB–dependent pathways, promoting osteoclastogenesis and increasing bone resorption. Under certain pathological conditions, inflammatory mediators may also transiently stimulate osteoblast activity, as observed in some rheumatic diseases; however, chronic inflammation generally favors net bone loss [82,83].

AFB_1_ induces inflammatory responses through multiple, interconnected mechanisms. One well-described pathway involves activation of Toll-like receptor 2 (TLR2), which recruits intracellular signaling cascades terminating in the nuclear translocation of activator protein-1 (AP-1) and NF-κB, leading to the transcriptional upregulation of pro-inflammatory cytokines [84]. AFB_1_ exposure has been shown to increase circulating and tissue levels of cytokines such as TNF-α and IL-6, reinforcing a pro-inflammatory milieu [84]. In addition, AFB_1_-induced oxidative stress acts as a potent upstream driver of inflammatory signaling, as ROS activate redox-sensitive transcription factors, including NF-κB, thereby linking oxidative damage to sustained inflammation. Although the direct impact of AFB_1_-induced inflammation on fetal skeletal development is not fully elucidated, substantial evidence indicates that inflammatory cytokines adversely affect bone growth during development. IL-1β and TNF-α act synergistically to impair longitudinal bone growth by exerting cytotoxic effects on growth plate chondrocytes and inhibiting matrix synthesis [85]. Moreover, overexpression of IL-6 has been shown to suppress osteoblast activity while enhancing osteoclast differentiation, leading to osteopenia and growth plate disorganization [86]. Taken together, AFB_1_-induced oxidative stress and inflammation converge to disrupt bone development through both maternal-mediated mechanisms, such as impaired calcium availability and endocrine dysfunction, and direct cellular toxicity affecting osteoblasts, osteoclasts, and chondrocytes. Recent experimental investigations have begun to provide more direct evidence of skeletal-specific consequences of AFB_1_ exposure, partly addressing the predominance of hepatic toxicity models. In vivo studies demonstrate that AFB_1_ impairs bone mineralization, reduces bone mineral density, and alters structural integrity in developing animals, including decreases in tibial ash content, bone length, and mechanical strength in poultry models, as well as attenuation of bone mass improvement in murine osteoporosis contexts [87,88,89]. Although these findings support a direct osteotoxic potential of AFB_1_ in addition to systemic oxidative and inflammatory mechanisms, dedicated investigations of osteoblast-, osteoclast-, and chondrocyte-specific molecular responses remain scarce, and human evidence is largely limited to indirect developmental outcomes rather than skeletal biomarkers. This imbalance highlights an important research gap and underscores the need for recent, bone-focused experimental and translational studies.

### 2.8. Evidence Strength and Translational Limitations

Prenatal AFB_1_-associated skeletal toxicity requires interpretation explicitly considering the hierarchical strength and translational limitations of the available evidence. Up-to-date mechanistic understanding derives predominantly from controlled animal experiments and in vitro studies, which enable manipulation of exposure timing, dosage, and molecular endpoints but do not fully reproduce human placental physiology, maternal–fetal toxicokinetics, or developmental timing [33,76]. Nevertheless, human evidence remains limited and is largely limited to epidemiological associations between maternal exposure biomarkers and nonspecific outcomes, including reduced birth weight and impaired growth trajectories [11,12,13]. Direct evidence of the fetal skeletal development, which can include ossification status, bone mineral density, or biochemical markers of bone turnover, are rarely available in human cohorts, so skeletal mechanistic conclusions rely heavily on biological likelihood inferred from experimental systems [48].

A major translational limitation is interspecies variability in hepatic metabolic capacity, placental structure and transport mechanisms, gestational duration, and the timing of skeletal maturation, which collectively influence fetal dose, detoxification efficiency, and susceptibility of osteogenic tissues during critical windows [33,57]. Moreover, many experimental studies employ exposure levels that exceed typical environmental dietary exposure, potentially expanding toxicological indications relative to chronic low-dose exposure patterns in human populations [21,33]. Cellular mechanistic studies add further uncertainty because they may rely on non-skeletal or transformed cell models and simplified oxidative-stress paradigms that cannot capture maternal–placental–fetal interactions in vivo [68,75]. These limitations underscore the need for integrated translational research combining physiologically relevant exposure models, standardized skeletal endpoints, comparative toxicokinetic approaches, and longitudinal human cohort studies incorporating skeletal-specific biomarkers to refine developmental risk assessment and improve clinical relevance [13,33,57]. Additional interspecies variability arises from differences in placental architecture (e.g., hemochorial versus endotheliochorial organization) and cytochrome P450 isoform expression, both of which influence maternal–fetal toxicokinetics and fetal tissue exposure. These biological distinctions further complicate quantitative extrapolation of skeletal risk from experimental models to human pregnancy. Experimental studies must focus on low doses that reflect real human dietary exposure. A key priority is characterizing toxicokinetics to understand how maternal intake affects the fetus. Physiologically Based Pharmacokinetic (PBPK) models are essential tools for this purpose [90].

Beyond simple morphology, bone development research should incorporate bone turnover markers (BTMs) measurable in fetal and umbilical cord blood. Monitoring formation markers, such as P1NP and Osteocalcin, and resorption markers, such as CTX-I, helps identify specific defects in osteoblast activity and mineralization [91]. Additionally, imaging methods like Quantitative Ultrasound (QUS) provide a non-invasive clinical parallel to experimental bone diaphanization. While diaphanization reveals the extent of ossified areas in animal models, QUS measures both the size and density of these regions in children [92]. This approach, combined with specific biomarkers, can determine the timing of ossification relative to pre- and postnatal exposure levels. Integrating these imaging and molecular tools provides a robust framework for assessing developmental skeletal toxicity, establishing a strong translational interface that allows researchers to validate animal findings in human clinical studies.

## 3. Conclusions and Perspectives

Bone alterations represent one of the most consistent and reproducible outcomes observed in experimental models of prenatal AFB_1_ exposure. Nevertheless, the molecular and physiological mechanisms underlying these skeletal abnormalities remain incompletely defined, reflecting the multifactorial and developmentally sensitive nature of AFB_1_ toxicity during gestation. The evidence synthesized in this review supports an integrated pathogenic framework in which disruption of vitamin D metabolism and parathyroid hormone–dependent calcium homeostasis reduces mineral availability for ossification, while oxidative stress and inflammatory signaling impair osteoblast, osteoclast, and chondrocyte function. These processes operate concurrently at maternal, placental, and fetal levels, where maternal hepatic, endocrine, and metabolic disturbances may amplify direct fetal toxicity following transplacental exposure. These converging pathways provide a mechanistic basis for delayed ossification, reduced mineralization, and structural skeletal abnormalities linked with prenatal AFB_1_ exposure. The findings based on the synthesized studies suggest that future research should move beyond descriptive teratology toward mechanistically resolved and translationally relevant investigation. Priority directions should comprise (i) cell-type–specific analyses of osteoblast, osteoclast, and growth-plate chondrocyte responses to AFB_1_ using molecular, transcriptomic, and epigenetic approaches, (ii) definition of critical gestational exposure windows and environmentally relevant dose–response relationships through integrated maternal–placental–fetal experimental designs, (iii) identification of sensitive biomarkers of early skeletal disruption in human populations, including imaging-based bone mineralization metrics and circulating bone-turnover markers, and (iv) development and validation of preventive strategies, such as dietary exposure reduction, improved food-storage practices, nutritional modulation of antioxidant and mineral status, and safe mycotoxin-binding interventions during pregnancy, particularly in regions with high aflatoxin burden. In the same way, translational research should address interspecies differences in metabolism, placental transfer, and skeletal maturation to improve extrapolation from animal models to human risk assessment. Integration of toxicokinetic modeling, longitudinal cohort studies, and mechanistic experimentation will be essential for establishing causal relationships between prenatal exposure and developmental bone outcomes. Based on current mechanistic evidence, several testable hypotheses emerge: (i) prenatal AFB_1_ exposure suppresses osteoblast differentiation through oxidative-stress–mediated disruption of RUNX2-dependent transcription; (ii) mid-gestation represents a critical susceptibility window due to peak chondrocyte hypertrophy and primary ossification center formation; and (iii) placental oxidative stress reduces fetal bone mineral accrual independently of maternal serum calcium concentration. Experimental validation of these hypotheses would substantially advance causal understanding of AFB_1_-associated skeletal toxicity. Progress in these research directions will not only clarify the biological basis of AFB_1_-induced skeletal toxicity but also inform evidence-based public-health strategies aimed at preventing mycotoxin-associated developmental disorders in vulnerable populations.

## Figures and Tables

**Table 1:** Effects of aflatoxin B_1_ (AFB_1_) on fetal bone development in different animal models.

Animal	Dose (mg/kg/day)	Exposure Route/Vehicle	Gestational Period (GD)	Skeletal Evaluation Method	Effects on Bone Development	Ref.
Rabbits *	0.025–0.1	Oral/corn oil	GD 6–18	Histological serial fetal sections (microscopic evaluation)	Cranial ossification defects and orbital enlargement under combined mycotoxin exposure	[10]
Rabbits	0.05	Gastric intubation/corn oil	GD 6–18; fetuses examined GD 29	Double skeletal staining (Alizarin Red-S/Alcian Blue), stereomicroscopy, mineralized length measurement	Incomplete ossification of skull, vertebrae, sternum, and limb elements	[44]
Rabbits	0.05 and 0.1	Gastric intubation/corn oil	GD 6–21 (dose-dependent fetal loss before term at high dose)	Double skeletal staining (Alizarin Red-S/Alcian Blue), mineralization assessment	Marked reduction in ossification and skeletal malformations, severity dose-dependent	[45]
Mice	20 (single dose)	Intraperitoneal injection	GD 7 or 13; fetuses examined GD 18	Skeletal malformation assessment of axial/appendicular ossification	Axial and appendicular hypoplasia, delayed supraoccipital ossification, cervical ribs, sternal	[46]
Rats	1	Oral gavage	GD 6–15; dams sacrificed GD 20	Double skeletal staining (Alizarin Red-S/Alcian Blue), histopathology, cytogenetics	Failure of ossification in skull, limbs, and spine; vertebral and limb defects	[47]
Rats *	0.125–1.0	Gastric intubation/corn oil	GD 6–15	Gross, skeletal, and visceral anomaly assessment; fetal growth indices	Dose-related fetal malformations and reduced fetal growth	[48]

* Combined exposure to ochratoxin A and aflatoxin B_1_ (OTA + AFB_1_). GD: gestational day; Alizarin Red S/Alcian Blue: differential staining method for mineralized bone and cartilage.

## Data Availability

The original contributions presented in this study are included in the article. Further inquiries can be directed to the corresponding authors.

## References

[1] Krasevec J., Blencowe H., Coffey C., Okwaraji Y.B., Estevez D., Stevens G.A., Ohuma E.O., Conkle J., Gatica-Domínguez G., Bradley E. (2022). Study Protocol for UNICEF and WHO Estimates of Global, Regional, and National Low Birthweight Prevalence for 2000 to 2020. Gates Open Res..

[2] Magnus M.C., Wilcox A.J., Morken N.-H., Weinberg C.R., Håberg S.E. (2019). Role of Maternal Age and Pregnancy History in Risk of Miscarriage: Prospective Register Based Study. BMJ.

[3] Kyei N.N.A., Boakye D., Gabrysch S. (2020). Maternal mycotoxin exposure and adverse pregnancy outcomes: A systematic review. Mycotoxin Res..

[4] Cotty P.J., Jaime-Garcia R. (2007). Influences of Climate on Aflatoxin Producing Fungi and Aflatoxin Contamination. Int. J. Food Microbiol..

[5] Kumar P., Mahato D.K., Kamle M., Mohanta T.K., Kang S.G. (2017). Aflatoxins: A Global Concern for Food Safety, Human Health and Their Management. Front. Microbiol..

[6] Benkerroum N. (2020). Aflatoxins: Producing-Molds, Structure, Health Issues and Incidence in Southeast Asian and Sub-Saharan African Countries. Int. J. Environ. Res. Public Health.

[7] Partanen H.A., El-Nezami H.S., Leppänen J.M., Myllynen P.K., Woodhouse H.J., Vähäkangas K.H. (2010). Aflatoxin B1 Transfer and Metabolism in Human Placenta. Toxicol. Sci..

[8] Kihara T. (2000). Effects of Prenatal Aflatoxin B1 Exposure on Behaviors of Rat Offspring. Toxicol. Sci..

[9] Supriya C., Reddy P.S. (2015). Prenatal Exposure to Aflatoxin B1: Developmental, Behavioral, and Reproductive Alterations in Male Rats. Sci. Nat..

[10] Wangikar P.B., Dwivedi P., Sinha N., Sharma A.K., Telang A.G. (2005). Teratogenic Effects in Rabbits of Simultaneous Exposure to Ochratoxin A and Aflatoxin B1 with Special Reference to Microscopic Effects. Toxicology.

[11] Andrews-Trevino J.Y., Webb P., Shively G., Rogers B.L., Baral K., Davis D., Paudel K., Pokharel A., Shrestha R., Wang J.-S. (2019). Relatively Low Maternal Aflatoxin Exposure Is Associated with Small-for-Gestational-Age but not with Other Birth Outcomes in a Prospective Birth Cohort Study of Nepalese Infants. J. Nutr..

[12] Lauer J.M., Duggan C.P., Ausman L.M., Griffiths J.K., Webb P., Wang J., Xue K.S., Agaba E., Nshakira N., Ghosh S. (2019). Maternal Aflatoxin Exposure during Pregnancy and Adverse Birth Outcomes in Uganda. Matern. Child Nutr..

[13] Fermiano J.T.A., Ali S., Ullah S., Rezende V.T., Rosim R.E., Tonin F.G., Ferri W.A.G., Marcolin A.C., Ramalho L.N.Z., Oliveira C.A.F.d. (2025). Assessment of Maternal Exposure to Mycotoxins During Pregnancy Through Biomarkers in Fetal and Neonatal Tissues. Toxins.

[14] Afriyeni H., Yosmar R., Rizal R., Fikri Z.A. (2025). Uji Efek Teratogenik Infusa Daun Kopi Arabika (*Coffea arabica* L.) Terhadap Fetus Mencit. J. Penelit. Dan Pengkaj. Ilm. Eksakta.

[15] Wangikar P.B., Dwivedi P., Sinha N., Sharma A.K., Telang A.G. (2005). Effects of Aflatoxin B1 on Embryo Fetal Development in Rabbits. Food Chem. Toxicol..

[16] Pickova D., Ostry V., Malir F. (2021). A Recent Overview of Producers and Important Dietary Sources of Aflatoxins. Toxins.

[17] Ali S., Freire L., Rezende V., Noman M., Ullah S., Abdullah, Badshah G., Afridi M., Tonin F., de Oliveira C. (2023). Occurrence of Mycotoxins in Foods: Unraveling the Knowledge Gaps on Their Persistence in Food Production Systems. Foods.

[18] Gemede H.F. (2025). Toxicity, Mitigation, and Chemical Analysis of Aflatoxins and Other Toxic Metabolites Produced by Aspergillus: A Comprehensive Review. Toxins.

[19] Syraji Y., Jeyaramraja P.R., Mada T., Gobikanila K. (2025). Comprehensive review of aflatoxin contamination, its occurrence, effects, management, and future perspectives. Discov. Food.

[20] Gorain S., Validandi V., Kurella S., Sagubandi Y., Sinha S.N. (2025). Aflatoxin exposure during pregnancy or infancy and its effect on infant health: A narrative review. Br. J. Nutr..

[21] Williams J.H., Phillips T.D., Jolly P.E., Stiles J.K., Jolly C.M., Aggarwal D. (2004). Human Aflatoxicosis in Developing Countries: A Review of Toxicology, Exposure, Potential Health Consequences, and Interventions. Am. J. Clin. Nutr..

[22] Marchese S., Polo A., Ariano A., Velotto S., Costantini S., Severino L. (2018). Aflatoxin B1 and M1: Biological Properties and Their Involvement in Cancer Development. Toxins.

[23] Lewis L., Onsongo M., Njapau H., Schurz-Rogers H., Luber G., Kieszak S., Nyamongo J., Backer L., Dahiye A.M., Misore A. (2005). Aflatoxin Contamination of Commercial Maize Products during an Outbreak of Acute Aflatoxicosis in Eastern and Central Kenya. Environ. Health Perspect..

[24] IARC Agents Classified by the IARC Monographs, Volumes 1–138—IARC Monographs on the Identification of Carcinogenic Hazards to Humans. https://monographs.iarc.who.int/agents-classified-by-the-iarc/.

[25] Hamid A.S., Tesfamariam I.G., Zhang Y., Zhang Z.G. (2013). Aflatoxin B1-Induced Hepatocellular Carcinoma in Developing Countries: Geographical Distribution, Mechanism of Action and Prevention. Oncol. Lett..

[26] Mary V.S., Theumer M.G., Arias S.L., Rubinstein H.R. (2012). Reactive Oxygen Species Sources and Biomolecular Oxidative Damage Induced by Aflatoxin B1 and Fumonisin B1 in Rat Spleen Mononuclear Cells. Toxicology.

[27] Aguilar F., Hussain S.P., Cerutti P. (1993). Aflatoxin B1 Induces the Transversion of G-->T in Codon 249 of the P53 Tumor Suppressor Gene in Human Hepatocytes. Proc. Natl. Acad. Sci. USA.

[28] El-Bahr S.M. (2015). Effect of Curcumin on Hepatic Antioxidant Enzymes Activities and Gene Expressions in Rats Intoxicated with Aflatoxin B1. Phytother. Res..

[29] Singh K.B., Maurya B.K., Trigun S.K. (2015). Activation of Oxidative Stress and Inflammatory Factors Could Account for Histopathological Progression of Aflatoxin-B1 Induced Hepatocarcinogenesis in Rat. Mol. Cell. Biochem..

[30] Lyman B.A., Erki L., Biedrzycka D.W., Devlin T.M., Ch’ih J.J. (1988). Modification of Protein Synthetic Components by Aflatoxin B1. Biochem. Pharmacol..

[31] Supriya C., Akhila B., Pratap Reddy K., Girish B.P., Sreenivasula Reddy P. (2016). Effects of Maternal Exposure to Aflatoxin B1 during Pregnancy on Fertility Output of Dams and Developmental, Behavioral and Reproductive Consequences in Female Offspring Using a Rat Model. Toxicol. Mech. Methods.

[32] Turner P.C. (2013). The Molecular Epidemiology of Chronic Aflatoxin Driven Impaired Child Growth. Scientifica.

[33] Smith L.E., Prendergast A.J., Turner P.C., Humphrey J.H., Stoltzfus R.J. (2017). Aflatoxin Exposure During Pregnancy, Maternal Anemia, and Adverse Birth Outcomes. Am. Soc. Trop. Med. Hyg..

[34] da Silva J.V.B., de Oliveira C.A.F., Ramalho L.N.Z. (2021). Effects of Prenatal Exposure to Aflatoxin B1: A Review. Molecules.

[35] Francis S., Kortei N.K., Sackey M., Richard S. (2024). Aflatoxin B1 induces infertility, fetal deformities, and potential therapies. Open Med..

[36] Tesfamariam K., Plekhova V., Gebreyesus S.H., Lachat C., Alladio E., Argaw A., Endris B.S., Roro M., De Saeger S., Vanhaecke L. (2024). Rapid LA-REIMS-Based Metabolic Fingerprinting of Serum Discriminates Aflatoxin-Exposed from Non-Exposed Pregnant Women: A Prospective Cohort from the Butajira Nutrition, Mental Health, and Pregnancy (BUNMAP) Study in Rural Ethiopia. Mycotoxin Res..

[37] Tesfamariam K., Argaw A., Hanley-Cook G.T., Gebreyesus S.H., Kolsteren P., Belachew T., Van de Velde M., De Saeger S., De Boevre M., Lachat C. (2022). Multiple Mycotoxin Exposure during Pregnancy and Risks of Adverse Birth Outcomes: A Prospective Cohort Study in Rural Ethiopia. Environ. Int..

[38] Tani S., Chung U., Ohba S., Hojo H. (2020). Understanding Paraxial Mesoderm Development and Sclerotome Specification for Skeletal Repair. Exp. Mol. Med..

[39] Noden D.M., Trainor P.A. (2005). Relations and Interactions between Cranial Mesoderm and Neural Crest Populations. J. Anat..

[40] Shahi M., Peymani A., Sahmani M. (2017). Regulation of Bone Metabolism. Rep. Biochem. Mol. Biol..

[41] Cardoso H.F.V. (2008). Age Estimation of Adolescent and Young Adult Male and Female Skeletons II, Epiphyseal Union at the Upper Limb and Scapular Girdle in a Modern Portuguese Skeletal Sample. Am. J. Phys. Anthropol..

[42] Hautier L., Charles C., Asher R.J., Gaunt S.J. (2014). Ossification Sequence and Genetic Patterning in the Mouse Axial Skeleton. J. Exp. Zool. B Mol. Dev. Evol..

[43] Mackie E.J., Ahmed Y.A., Tatarczuch L., Chen K.-S., Mirams M. (2008). Endochondral Ossification: How Cartilage Is Converted into Bone in the Developing Skeleton. Int. J. Biochem. Cell Biol..

[44] Berendsen A.D., Olsen B.R. (2015). Bone Development. Bone.

[45] El-Nahla S., Imam H., Moussa E., Ibrahim A., Ghanam A. (2013). Teratogenic Effects of Aflatoxin in Rabbits (*Oryctolagus cuniculus*). J. Vet. Anat..

[46] Abdulrazzaq Y.M., Padmanabhan R., Bastaki S., Kochyil J., Shafiullah M. (2011). Teratogenic Effects of Aflatoxin B1 in Mice Exposed in Early and Late Gestation. Pediatr. Res..

[47] Fetaih H.A., Dessouki A.A., Hassanin A.A.I., Tahan A.S. (2014). Toxopathological and Cytogenetic Effects of Aflatoxin B1 (AFB1) on Pregnant Rats. Pathol. Res. Pract..

[48] Wangikar P.B., Dwivedi P., Sinha N. (2004). Effect in Rats of Simultaneous Prenatal Exposure to Ochratoxin A and Aflatoxin B_1_. I. Maternal Toxicity and Fetal Malformations. Birth Defects Res. B Dev. Reprod. Toxicol..

[49] Ominsky M.S., Stouch B., Schroeder J., Pyrah I., Stolina M., Smith S.Y., Kostenuik P.J. (2011). Denosumab, a Fully Human RANKL Antibody, Reduced Bone Turnover Markers and Increased Trabecular and Cortical Bone Mass, Density, and Strength in Ovariectomized Cynomolgus Monkeys. Bone.

[50] Saponaro F., Saba A., Zucchi R. (2020). An Update on Vitamin D Metabolism. Int. J. Mol. Sci..

[51] Fleet J.C. (2022). Vitamin D-Mediated Regulation of Intestinal Calcium Absorption. Nutrients.

[52] Rillaerts K., Verlinden L., Doms S., Carmeliet G., Verstuyf A. (2025). A Comprehensive Perspective on the Role of Vitamin D Signaling in Maintaining Bone Homeostasis: Lessons from Animal Models. J. Steroid Biochem. Mol. Biol..

[53] Sergeev I.N., Arkhapchev I.P., Kravchenko L.V., Kodentsova V.M., Piliia N.M. (1988). Effect of Mycotoxins Aflatoxin B1 and T-2 Toxin on the Vitamin D3 Metabolism and Binding of Its Hormonal Form 1,25-Dihydroxyvitamin D3 in Rats. Vopr. Meditsinskoi Khimii.

[54] Costanzo P., Santini A., Fattore L., Novellino E., Ritieni A. (2015). Toxicity of Aflatoxin B1 towards the Vitamin D Receptor (VDR). Food Chem. Toxicol..

[55] Glahn R.P., Beers K.W., Bottje W.G., Wideman R.F., Huff W.E., Thomas W. (1991). Aflatoxicosis Alters Avian Renal Function, Calcium, and Vitamin d Metabolism. J. Toxicol. Environ. Health.

[56] Nassar A.Y., Galal A.F., Mohamed M.A., Megalla S.E., Hafez A.H. (1985). The Effect of Aflatoxin B1 on the Utilization of Serum Calcium. Mycopathologia.

[57] Stenhouse C., Suva L.J., Gaddy D., Wu G., Bazer F.W. (2022). Phosphate, Calcium, and Vitamin D: Key Regulators of Fetal and Placental Development in Mammals. Adv. Exp. Med. Biol..

[58] Zhou W., Duan T. (2023). Effects of Maternal Calcium and Protein Intake on the Development and Bone Metabolism of Offspring Mice. Open Life Sci..

[59] Lombardi G., Di Somma C., Rubino M., Faggiano A., Vuolo L., Guerra E., Contaldi P., Savastano S., Colao A. (2011). The Roles of Parathyroid Hormone in Bone Remodeling: Prospects for Novel Therapeutics. J. Endocrinol. Investig..

[60] Alexander R.T., Dimke H. (2023). Effects of Parathyroid Hormone on Renal Tubular Calcium and Phosphate Handling. Acta Physiol..

[61] Salcedo-Betancourt J.D., Moe O.W. (2024). The Effects of Acid on Calcium and Phosphate Metabolism. Int. J. Mol. Sci..

[62] Burton G.J., Jauniaux E. (2011). Oxidative Stress. Best Pract. Res. Clin. Obstet. Gynaecol..

[63] Grzeszczak K., Łanocha-Arendarczyk N., Malinowski W., Ziętek P., Kosik-Bogacka D. (2023). Oxidative Stress in Pregnancy. Biomolecules.

[64] Liu Y., Wang W. (2016). Aflatoxin B1 Impairs Mitochondrial Functions, Activates ROS Generation, Induces Apoptosis and Involves Nrf2 Signal Pathway in Primary Broiler Hepatocytes. Anim. Sci. J..

[65] Obidoa O., Obunwo C.C. (1979). Action of Aflatoxin on Some Redox Enzymes and Complexes of Avian Liver Mitochondria. Biochem. Med..

[66] Gopalan-Kriczky P., Hiruma S., Lotlikar P.D. (1994). Effect of Glutathione Levels on Aflatoxin B1-DNA Binding in Livers and Kidneys of Male Rats and Hamsters Pretreated with Buthionine Sulfoximine and Diethylmaleate. Cancer Lett..

[67] Ankrah N.-A., Sittie A., Addo P.G.A., Ekuban F.A. (1995). Enhanced Depletion of Glutathione and Increased Liver Oxidative Damage in Aflatoxin-Fed Mice Infected with Plasmodium Berghei. Trans. R. Soc. Trop. Med. Hyg..

[68] Ma J., Liu Y., Guo Y., Ma Q., Ji C., Zhao L. (2021). Transcriptional Profiling of Aflatoxin B1-Induced Oxidative Stress and Inflammatory Response in Macrophages. Toxins.

[69] Tadee A., Mahakunakorn P., Porasuphatana S. (2020). Oxidative Stress and Genotoxicity of Co-Exposure to Chlorpyrifos and Aflatoxin B 1 in HepG2 Cells. Toxicol. Ind. Health.

[70] Bădilă A.E., Rădulescu D.M., Ilie A., Niculescu A.-G., Grumezescu A.M., Rădulescu A.R. (2022). Bone Regeneration and Oxidative Stress: An Updated Overview. Antioxidants.

[71] Almeida M., Han L., Ambrogini E., Bartell S.M., Manolagas S.C. (2010). Oxidative Stress Stimulates Apoptosis and Activates NF-ΚB in Osteoblastic Cells via a PKCβ/P66shc Signaling Cascade: Counter Regulation by Estrogens or Androgens. Mol. Endocrinol..

[72] Kousteni S. (2011). FoxOs: Unifying Links Between Oxidative Stress and Skeletal Homeostasis. Curr. Osteoporos. Rep..

[73] Ambrogini E., Almeida M., Martin-Millan M., Paik J.-H., DePinho R.A., Han L., Goellner J., Weinstein R.S., Jilka R.L., O’Brien C.A. (2010). FoxO-Mediated Defense against Oxidative Stress in Osteoblasts Is Indispensable for Skeletal Homeostasis in Mice. Cell Metab..

[74] Zhang Y., Xiong Y., Zhou J., Xin N., Zhu Z., Wu Y. (2018). FoxO1 Expression in Osteoblasts Modulates Bone Formation through Resistance to Oxidative Stress in Mice. Biochem. Biophys. Res. Commun..

[75] Ha H., Bok Kwak H., Woong Lee S., Mi Jin H., Kim H.-M., Kim H.-H., Hee Lee Z. (2004). Reactive Oxygen Species Mediate RANK Signaling in Osteoclasts. Exp. Cell Res..

[76] Frank L., Ilene Sosenko R.S. (1987). Prenatal Development of Lung Antioxidant Enzymes in Four Species. J. Pediatr..

[77] Zima T., Štípek S., Crkovská J., Doudová D., Měchurová A., Calda P. (1996). Activity of the Antioxidant Enzymes Superoxide Dismutase and Glutathione Peroxidase in Fetal Erythrocytes. Prenat. Diagn..

[78] Dennery P.A. (2007). Effects of Oxidative Stress on Embryonic Development. Birth Defects Res. C Embryo Today.

[79] Prater M.R., Laudermilch C.L., Liang C., Holladay S.D. (2008). Placental Oxidative Stress Alters Expression of Murine Osteogenic Genes and Impairs Fetal Skeletal Formation. Placenta.

[80] Neumann E., Müller-Ladner U., Frommer K.W. (2014). Inflammation and Bone Metabolism. Z. Rheumatol..

[81] Lange U., Teichmann J., Schett G., Neumann E., Müller-Ladner U. (2013). Osteoimmunological Aspects on Inflammation and Bone Metabolism. J. Rheum. Dis. Treat.

[82] Baum R., Gravallese E.M. (2014). Impact of Inflammation on the Osteoblast in Rheumatic Diseases. Curr. Osteoporos. Rep..

[83] Jimi E., Takakura N., Hiura F., Nakamura I., Hirata-Tsuchiya S. (2019). The Role of NF-ΚB in Physiological Bone Development and Inflammatory Bone Diseases: Is NF-ΚB Inhibition “Killing Two Birds with One Stone”?. Cells.

[84] Radzka-Pogoda A., Radzki R.P., Bieńko M., Szponar J., Sokołowska B., Kulik A., Lewicka M., Borzęcki A. (2023). Ochratoxin A and Aflatoxin B1 as Factors of Bone Damage and Neurodegeneration Through the Influence on the Immunomodulation Processes of TNF-α and IL-6 Concentrations. Pol. Hyperb. Res..

[85] Mårtensson K., Chrysis D., Sävendahl L. (2004). Interleukin-1β and TNF-α Act in Synergy to Inhibit Longitudinal Growth in Fetal Rat Metatarsal Bones. J. Bone Miner. Res..

[86] De Benedetti F., Rucci N., Del Fattore A., Peruzzi B., Paro R., Longo M., Vivarelli M., Muratori F., Berni S., Ballanti P. (2006). Impaired Skeletal Development in Interleukin-6–Transgenic Mice: A Model for the Impact of Chronic Inflammation on the Growing Skeletal System. Arthritis Rheum..

[87] Paneru D., Sharma M.K., Shi H., Wang J., Kim W.K. (2024). Aflatoxin B1 Impairs Bone Mineralization in Broiler Chickens. Toxins.

[88] Mesgar A., Aghdam Shahryar H., Bailey C.A., Ebrahimnezhad Y., Mohan A. (2022). Effect of Dietary L-Threonine and Toxin Binder on Performance, Blood Parameters, and Immune Response of Broilers Exposed to Aflatoxin B1. Toxins.

[89] Lu S., Yuan Q., Wang L., Su D., Hu M., Guo L., Kang C., Zhou T., Zhang J. (2025). Aflatoxin B1 Contamination Reduces the Saponins Content and Anti-Osteoporosis Efficacy of the Traditional Medicine Radix Dipsaci. J. Ethnopharmacol..

[90] Allegaert K., Quinney S.K., Dallmann A. (2024). Physiologically Based Pharmacokinetic Modeling in Pregnancy, during Lactation and in Neonates: Achievements, Challenges and Future Directions. Pharmaceutics.

[91] D’Amato G., Brescia V., Fontana A., Natale M.P., Lovero R., Varraso L., Di Serio F., Simonetti S., Muggeo P., Faienza M.F. (2024). Biomarkers and Biochemical Indicators to Evaluate Bone Metabolism in Preterm Neonates. Biomedicines.

[92] Baroncelli G.I. (2008). Quantitative Ultrasound Methods to Assess Bone Mineral Status in Children: Technical Characteristics, Clinical Application, and Future Perspectives. Pediatr. Res..

